# Modeling of Thermal Conductivity of CVI-Densified Composites at Fiber and Bundle Level

**DOI:** 10.3390/ma9121011

**Published:** 2016-12-13

**Authors:** Kang Guan, Jianqing Wu, Laifei Cheng

**Affiliations:** 1 School of Materials Science and Engineering, South China University of Technology, Guangzhou 510640, China; imjqwu@scut.edu.cn; 2 Science and Technology on Thermostructural Composite Materials Laboratory, Northwestern Polytechnical University, Xi’an 710072, China; chenglf@nwpu.edu.cn

**Keywords:** thermal conductivity, ceramic matrix composites, chemical vapor infiltration, porosity, cracking, interface

## Abstract

The evolution of the thermal conductivities of the unidirectional, 2D woven and 3D braided composites during the CVI (chemical vapor infiltration) process have been numerically studied by the finite element method. The results show that the dual-scale pores play an important role in the thermal conduction of the CVI-densified composites. According to our results, two thermal conductivity models applicable for CVI process have been developed. The sensitivity analysis demonstrates the parameter with the most influence on the CVI-densified composites’ thermal conductivity is matrix cracking’s density, followed by volume fraction of the bundle and thermal conductance of the matrix cracks, finally by micro-porosity inside the bundles and macro-porosity between the bundles. The obtained results are well consistent with the reported data, thus our models could be useful for designing the processing and performance of the CVI-densified composites.

## 1. Introduction

The chemical vapor infiltration (CVI) process has been considered as one of the most important fabrication methods for advanced thermostructural materials [1,2,3,4,5]. Compared with the conventional processing techniques, CVI techniques have distinct advantages, such as minimizing damage to fibers, imposing little mechanical stress on fibers, applicable for a wide variety of ceramic matrix composite materials, producing near-net-shape composites. Despite these advantages, they also have some drawbacks, such as the long processing time, premature closing of the pores at the surface. For fabricating the thick composites by less time, the forced flow (F-CVI) and thermal gradient chemical vapor infiltration (TG-CVI) process has been developed as alternatives to isothermal-isobaric chemical vapor infiltration (ICVI) [1,6]. In TG-CVI, a thermal gradient is imposed on the preform to prevent premature sealing of the gas entrance. Thus, the detailed knowledge of the evolution of the thermal transport properties of the preform during the infiltration is essential for understanding and optimizing TG-CVI.

For now, relatively few numerical studies [7,8,9,10] have been carried out on this problem. For instance, considering the fiber and matrix as the same material and pores as non-conductive, Tomadakis and Sotirchos [7,11] used Monte Carlo algorithm to calculate the thermal transport properties of 1-D partially overlapping and randomly overlapping fiber structure. In contrast, Skamser et al. [8] considered the contribution of void phase to the thermal transport properties and provided more realistic data for 1-D SiC_f_/SiC composite obtained by their simulated microstructure. Vignoles et al. [9] firstly computed the thermal conductivity for a square array of fibers coated with anisotropic pyrocarbon matrix, then estimated the thermal conductivity of stitched cloth layup composites according to the microstructure geometric information gained through X-ray computerized microtomography (CMT). To the best of our knowledge, very few numerical studies have been reported for thermal transport properties of 2D woven and 3D braided composites in the bundle scale for CVI process.

Most CVI-densified composites possess high temperature capability and have been used in a number of demanding applications in high temperature environments. Hence a knowledge of the thermal transport properties of such composites is also of considerable importance and has been the subject of extensive experimental [12,13,14,15,16,17] and theoretical [18,19,20] studies. However, although a lot of thermal conductivity models in the fiber and bundle scales have been developed, the one appropriate ones for CVI-densified composites are very few, due to the existence of the dual-scale pores inside the composites. In addition, CVI-densified composites may be fabricated depending on the fiber type selected and the chosen densification route. Hence it is important, not only to generate experimental data, but also to characterize the thermal transport behavior in terms of constituent properties across the wide range of porosity.

For the previous reasons, the present work is the continuation of our previous study [21,22] and aimed at developing an efficient thermal conductivity model for CVI-densified composites. To accomplish this, the unidirectional fiber structures, 2D woven and 3D braided bundle structures are taken as research subjects. The effect of the individual component (fiber, interfacial layer and pore) on the transverse thermal conductivity of unidirectional fiber structures is first numerically investigated. Secondly, we numerically simulate the evolution of the macro-pore structures for 2D and 3D composites and investigate the effect of the macro-pore on the thermal conductivity of the simulated structures. Finally, two analytical models for these structures are developed based on our numerical data and compared to the available experimental data.

## 2. Unidirectional Fiber Structures

### 2.1. Description of the Model

For unidirectional fiber structures, the thermal conductivity parallel to the fiber direction is considered to be well described by the rule of mixtures, while the situation in the transverse direction is more complicated. Thus, the purpose of this section is to develop an analytical model to relate the transverse thermal conductivity of these structures to the ones of their four components (fiber, interfacial layer, matrix and pore).

**Figure 1:**
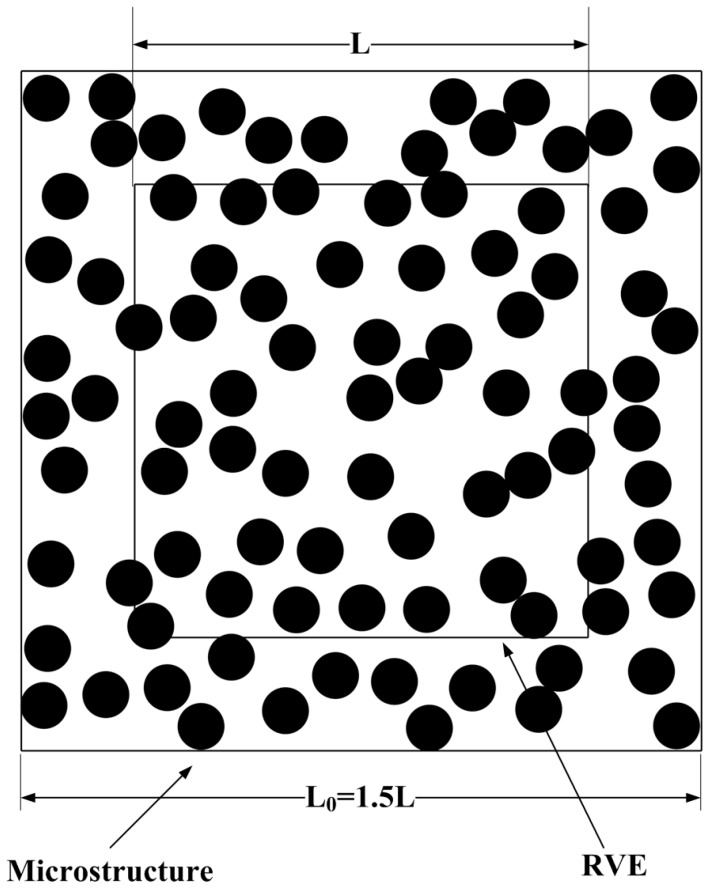
Depiction of RVE (representative volume element) for calculation of thermal conductivity.

To investigate the fiber distribution effects on transverse thermal conductivity of fiber-reinforced composites, the microstructure with random fiber distribution can be generated using a program developed in MATLAB. Specifically, the procedure starts with a specified microstructure with square cross-section and proceeds by randomly and sequentially adding the circular fiber inside the microstructure. Each added fiber is accepted and its position is recorded only if it does not overlap with any of the ones previously accepted. For high fiber volume fraction, a small amount of overlapping is allowable. In addition, the boundaries of the microstructure act like rigid walls; thus, no fibers pass through the boundaries, and all fibers are restricted inside the microstructure. The microstructures generated in this manner are primarily governed by the choice of porosity *p* and the allowable inter-fiber distance *d*_min_. The former is calculated as $p=1-\left(N_{f}\pi {r_{f}}^{2}\right)/{L_{0}}^{2}$, where *N_f_* is the number of included fibers, *r_f_* is the fiber radius, and *L*_0_ is the length of the microstructure. The latter (*d*_min_) is defined such that the shortest allowable distance between the centers of two fibers is larger than (2 + *d*_min_)*r_f_*. The selection values of *d*_min_ are 0.1 or 0 in this study. For the case of heat transfer transverse to the axis of randomly distributed fibers, the representative volume element (RVE) may be simulated by a square window placed on the cross section of the microstructure with the following relative scale dimensions: *L*_0_ = 1.5*L*, where *L* is the length of the RVE (as depicted in Figure 1). In addition, to assure the RVE contains a statistical representation of all local heterogeneities, the relative RVE size with respect to the scale of microstructural dimensions should be sufficiently large. The study of the scale effects indicates that the estimated thermal conductivities (as averaged over three independent simulation runs) are almost constant when the REV size is larger than 50*r_f_*, thus the sizes of RVE are set as 50*r_f_* × 50*r_f_* in this study.

To characterize the isotropy of the simulated fiber structures, we consider there are *N* fibers located less than distance *R* apart from the center of the microstructure, where *R* = 50*r_f_* in this study, and introduce the anisotropy parameter ($\bar{\phi }$):

(1) $$\bar{\phi }=\frac{\sum_{i=1}^{N}\text{cos}\;2\theta_{i}}{N}$$

where $\theta_{i}$ is the orientation angle (with respect to the positive x coordinate) of the line connecting the center of the unit cell and the *i*th fiber. The values of $\theta_{i}$ is +1 or −1 when all fibers are distributed along the x-axis or along the y-axis. When fibers structure is isotropic, $\bar{\phi }$ approaches zero. The absolute values of $\bar{\phi }$ were found typically less than 0.01 for all simulated fiber structures, which we consider to satisfy the isotropy requirement.

**Figure 2:**
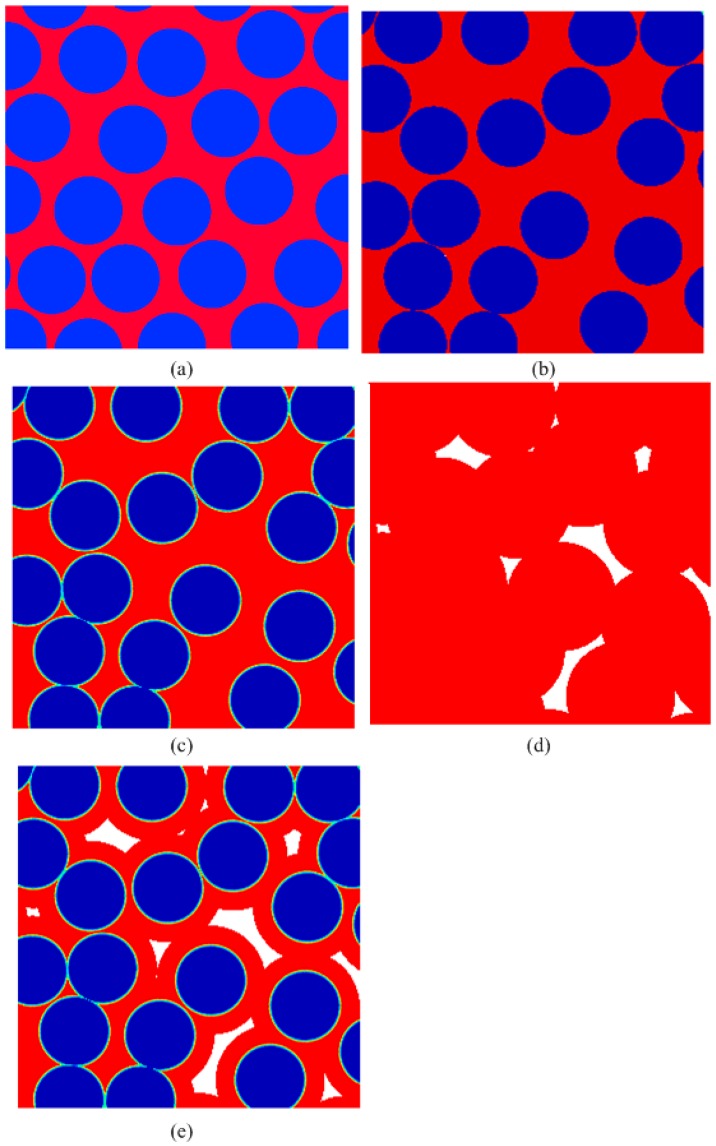
Illustration of the geometric models for unidirectional fiber structures: (**a**) The material systems composed of fiber and matrix with *d*_min_ = 0.1; The material systems with *d*_min_ = 0; (**b**) fiber and matrix; (**c**) fiber, interface and matrix; (**d**) matrix and pore; (**e**) all constituents.

**Figure 3:**
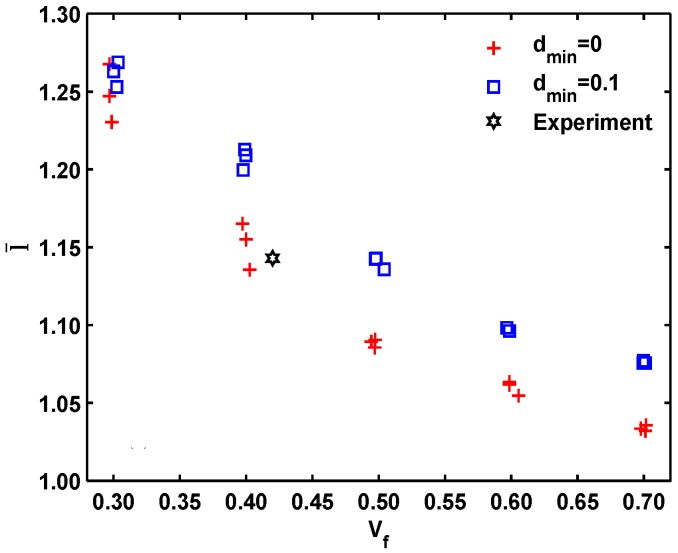
The normalized average nearest inter-fiber distance with the volume fraction of fiber.

The resultant microstructures are shown in Figure 2a,b. It can be observed that the generated fiber structures have different degree of local non-uniformity, although all of them are statistically homogenous. Thus, prior to any analysis, it is necessary to quantify the microstructure of the fiber distribution. In this effort, the normalized average nearest inter-fiber distance, $\bar{l}$, whose statistics differ between different fiber distributions, is introduced to characterize the degree of the non-uniformity. $\bar{l}$ is defined as

(2) $$\bar{l}=\left(\sum_{i}\frac{L_{i}}{r_{i}+r_{i}^{\text{nearest}}}\right)/N$$

where *L_i_* is the nearest distance between the *i*th fiber and its neighbor, *r_i_* is the radius of the *i*th fiber and *r_i_*^nearest^ is the radius of the fiber which is nearest to the *i*th fiber. Given the same fiber volume, small values of $\bar{l}$ are associated with heterogeneous patterns, while large values of $\bar{l}$ indicate homogeneous patterns, and its variation can be seen as a measure of the fiber dispersion. In Figure 3, the data for two fiber structures are compared with Spitzig’s experimental measurement [23]. We can observe that, Spitzig’s experimental data is consistent with our data for the simulated fiber structure with *d*_min_ = 0, hence only the thermal conductivities of the simulated fiber structure with *d*_min_ = 0 are chosen as the research subject.

By adopting the locations of the fibers and increasing the radius of the circles, the material systems with different phase have been developed and shown in Figure 2b–e. Appling the corresponding temperature and thermal insulation boundaries to the unit cells, the transverse thermal conductivity of these material systems can be calculated using finite element software “COMSOL Multiphysics” coupled with MATLAB. Then the effect of all constituent (fiber, interfacial layer, matrix and pore) thermal conductivities on the global thermal transport properties of the material systems can be studied. The full solving details can be found in Reference [24].

### 2.2. The Analytical Model

In 1892, Rayleigh [25] investigated the conductivity of an uniform medium composed of cylindrical obstacles (fibers) arranged in rectangular order, applied two boundary conditions (radial heat flux and temperature potential continuity) at the fiber/matrix interface and solved the steady-state heat conduction (mathematically the potential equation) to give approximate function to predict transverse ETC [26,27]:

(3) $$K_{e}^{T}/K_{m}\approx 1-2V_{f}/\left(V_{f}+B-0.3058{V_{f}}^{4}/B-0.0134{V_{f}}^{8}/B\right)$$

where *B* is a dimensionless variable defined as

(4) B=(1+k)/(1−k)

(5) $$k=K_{f}^{T}/K_{m}$$

where *V_f_*, $K_{e}^{T}$, *K_m_*, $K_{f}^{T}$ and *k* are the volume fraction of fiber, the transverse thermal conductivity of the one-dimensional composite, the thermal conductivity of the isotropic matrix, the transverse thermal conductivity of isotropic fiber and the fiber-to-matrix conductivity ratio, respectively. When the fiber volume fraction is low, the higher order terms of Equation (3) can be ignored, then Rayleigh’s model reduces to the so called self-consistent formula [28]:

(6) $$K_{e}^{T}/K_{m}=\left[k+1+\left(k-1\right)V_{f}\right]/\left[k+1-\left(k-1\right)V_{f}\right]$$

Another famous model is developed by Hasselman and Johnson [29], who considered the imperfect interface between fibers and matrix and introduced an effective interfacial conductance *H_i_*, their model is expressed as:

(7) $$K_{e}^{T}/K_{m}=\frac{\left[K_{f}^{T}/K_{m}-1-K_{f}^{T}/\left(r_{f}H_{i}\right)\right]V_{f}+\left[1+K_{f}^{T}/\left(r_{f}H_{i}\right)+K_{f}^{T}/K_{m}\right]}{\left[1+K_{f}^{T}/\left(r_{f}H_{i}\right)-K_{f}^{T}/K_{m}\right]V_{f}+\left[1+K_{f}^{T}/\left(r_{f}H_{i}\right)+K_{f}^{T}/K_{m}\right]}$$

where *r_f_* is the fiber radius. If we introduced an interfacial Biot number, *B_i_* ($B_{i}=H_{i}r_{f}/K_{f}^{T}$) and Equation (5) into Hasselman and Johnson’s model, then H-J model can be easily rearranged into the following form:

(8) $$K_{e}^{T}/K_{m}=\frac{k/\left(1+1/B_{i}\right)+1+\left[k/\left(1+1/B_{i}\right)-1\right]V_{f}}{k/\left(1+1/B_{i}\right)+1-\left[k/\left(1+1/B_{i}\right)-1\right]V_{f}}$$

Comparing Equation (6) with (8), it is clearly found that the self-consistent model is just the special case of H-J model when 1/*B_i_*→0, the composites with imperfect fiber/matrix interface can be practically seen as the ones with perfect conductance, in which the effect of imperfect interface is lumped into fiber-to-matrix conductivity ratio *k*. Typical to the cylindrically orthotropic carbon fibers-reinforced composites, Later, Hasselman [30] used the same method to develop the following formula to estimate the transverse thermal conductivity:

(9) $$K_{e}^{T}/K_{m}=\frac{\left[\lambda /K_{m}-1-\lambda /\left(r_{f}H_{i}\right)\right]V_{f}+\left[1+\lambda /\left(r_{f}H_{i}\right)+\lambda /K_{m}\right]}{\left[1+\lambda /\left(r_{f}H_{i}\right)-\lambda /K_{m}\right]V_{f}+\left[1+\lambda /\left(r_{f}H_{i}\right)+\lambda /K_{m}\right]}\;\text{with}\;\lambda ={\left(K_{\theta }K_{r}\right)}^{1/2}$$

where *K_θ_* and *K_r_* are the radial and tangential thermal conductivity of the cylindrically orthotropic fiber, respectively. Obviously, Equation (9) agrees to Equation (7) if $K_{\theta }=K_{r}=K_{f}^{T}$.

**Figure 4:**
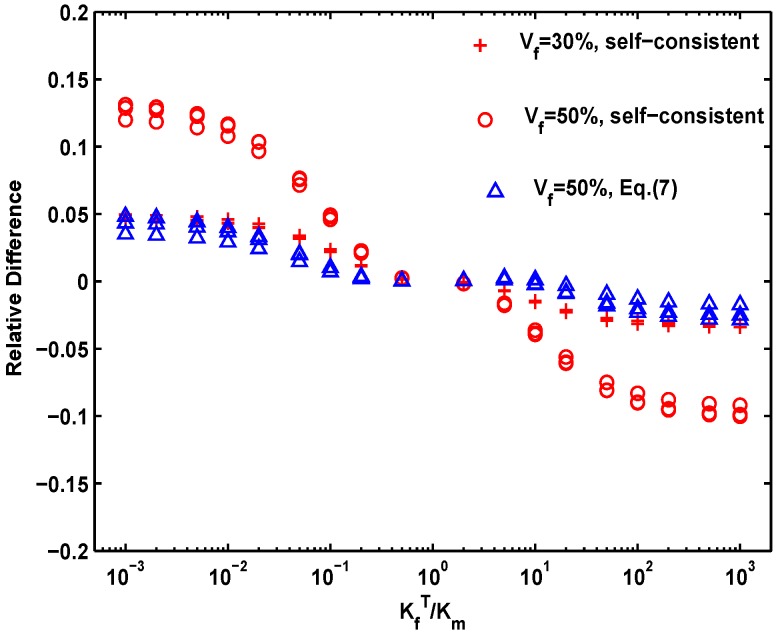
Comparison of the finite element results for effective thermal conductivity of the material systems composed of fiber and matrix with the analytical solution of the self-consistent (Equation (6)) and the fitted (Equation (11)) equations.

However, above models are developed on the assumption that the heat flux through the single fiber is not affected by the others, thus these developed formulae are valid only for dilute fiber concentrations. At higher volume-fractions of fiber, these formulae are approximate or even erroneous, for the local temperature fields around the fibers will interact. The differences between the calculated transverse thermal conductivity of two-phase model (as shown in Figure 2b) and the prediction of self-consistent formula with the fiber-to-matrix conductivity ratio are shown in Figure 4. The relative differences (RD) between both are illustrated by the following function:

(10) $$\text{Relative Difference}\left(\text{RD}\right)=\frac{K_{MOD}^{e}-K_{FEM}^{e}}{K_{FEM}^{e}}$$

where the subscripts MOD and FEM denote the *K^e^* values are calculated by the model and the simulation, respectively. It is found that the self-consistent formula can give a satisfactory prediction for all fiber structures when 0.01 < $K_{f}^{T}/K_{m}$ < 100. When $K_{f}^{T}/K_{m}$ < 0.01 or $K_{f}^{T}/K_{m}$ > 100, the following fitting function can be used:

(11) $$K_{e}^{T}/K_{m}=1-2V_{f}/\left(V_{f}+B-0.07{V_{f}}^{0.3}/B\right)$$

where *B* has been defined by Equation (4). In Figure 4, Equation (11) is shown to give a satisfactory agreement to our data. In most cases, the fiber-matrix ratio is at the range of 10^−2^–10^2^, thus the self-consistent formula (or Hasselman and Johnson’s model) is satisfactory for estimation.

**Figure 5:**
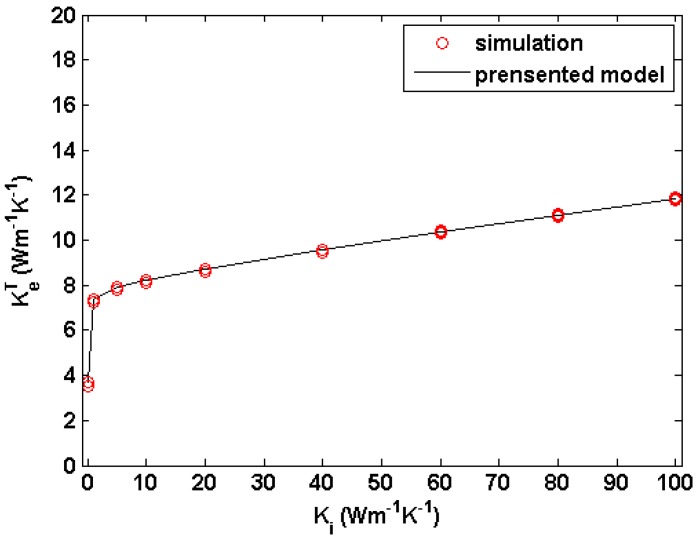
Comparison of the calculated transverse thermal conductivity of three-phase system and the predicted values by the presented model for $K_{f}^{T}$ = 3 W·m^−1^·K^−1^, *K_m_* = 20 W·m^−1^·K^−1^, *V_f_* = 0.5 and *V_i_* = 0.05. The three-phase system is composed of fiber, interface layer and matrix.

To improve the interface between matrix and fiber, the fibers are coated by the pyrolytic carbon (PyC) interfacial layer before the deposition of the matrix. In self-consistent (Equation (6)) and H-J model (Equation (7)), a fiber is in the middle of a cylindrical matrix and the matrix is contained within an effectively homogeneous medium, which is very close to the situation of CVI processing if the thickness of the interfacial layer is not too large. Then the coated fiber with interface layer can be approximately treated as a homogeneous solid phase, whose volume fraction is (*V_f_* + *V_i_*), where *V_i_* is the volume fraction of the interfacial layer. In addition, the equivalent transverse thermal conductivity of the solid phase, $K_{fi}^{T}$ can be estimated according to H-J model:

(12) $$K_{fi}^{T}=K_{i}\frac{\frac{K_{f}^{T}}{K_{i}}+1+\left(\frac{K_{f}^{T}}{K_{i}}-1\right)\left(\frac{V_{f}}{V_{f}+V_{i}}\right)}{\frac{K_{f}^{T}}{K_{i}}+1-\left(\frac{K_{f}^{T}}{K_{i}}-1\right)\left(\frac{V_{f}}{V_{f}+V_{i}}\right)}$$

where *K_i_* is the thermal conductivity of the interfacial layer. Substituting Equation (12) into (6), the transverse thermal conductivity of the three-phase system can be estimated. To validate this model, by setting $K_{f}^{T}$= 3 W·m^−1^·K^−1^, *K_m_* = 20 W·m^−1^·K^−1^, *V_f_* = 0.5 and *V_i_* = 0.05, the transverse thermal conductivity of the three-phase system (as shown in Figure 2c) with *K_i_* values are calculated and shown in Figure 5. It is clearly shown that the presented model gives a good fit to our simulation results.

Considering that the fiber, matrix and interfacial layer have the same thermal conductivity values, *K_s_*, above a certain pore concentration, the transverse thermal conductivities of the systems are the same order of magnitude as the thermal conductivity of the pore, which indicates that the pore phase is the dominant pathway for heat transfer. As the solid volume fraction is increased and larger than the ‘critical solid concentration’, $V_{s}^{c}$, the matrix deposited on the surfaces of the different fibers contact with each other and form a continuous path for heat transfer. Noted a parameter called the critical pore concentration has been frequently used to estimate the gas transport properties of the densified microstructure for CVI process, then the value of $V_{s}^{c}$ can be determined by subtracting the critical pore concentration for 1-D partially overlapping fiber structure from one, the following expression can be given from our simulation results:

(13) $$V_{s}^{c}=1-0.33{\left(1-V_{f}\right)}^{0.19}$$

A phase interchange theorem is developed by Keller [31] for two-dimensional statistically homogeneous, isotropic structures, which simply states that, the $K_{e}^{T}/K_{m}$ value of this structure by interchanging the thermal conductivity values of fiber and matrix is equal to the reciprocal of the $K_{e}^{T}/K_{m}$ value of the original structure. When *V_s_* is less than $V_{s}^{c}$, than Tomadakis and Sotirchos [32] applied Keller’s theorem and related the transverse thermal transport of 1-D fiber structure when the fiber are high-conductive and the gas transport of 1-D overlapping fiber structure when the fiber are non-conductive:

(14) $${\frac{K_{e}^{T}}{K_{p}}|}_{K_{s}>>K_{p}}=\frac{D}{D^{e}}=\frac{\tau }{V_{p}}$$

where ${\frac{K_{e}^{T}}{K_{p}}|}_{K_{s}>>K_{p}}$ is the reduce transverse thermal conductivity of the systems when the solid thermal conductivity is highly larger then the one of pore, τ is the Fick diffusion tortuosity of the systems [22].

Due to the local non-uniformity of the fiber structure, the coated fibers will overlap each other and form the local conductive chains. If the local conductive chains can be approximated as the fibers with elliptic cross-section, then the effective thermal conductivity of this material system can be estimated by Tsai-Halpin equations [33,34]:

(15) $$K_{e}^{T}/K_{p}=\left(1+\xi \eta V_{s}\right)/\left(1-\eta V_{s}\right)\;\text{with}\;\eta =\left(K_{s}/K_{p}-1\right)/\left(K_{s}/K_{p}+\xi \right)$$

for elliptic fibers with aspect ratios *e*, $\xi =\sqrt{3}\;\text{ln}\left(e\right)$. Then the reduced transverse thermal conductivity of the systems can be obtained by Tsai-Halpin equations when the solid is high-conductive:

(16) $${\frac{K_{e}^{T}}{K_{p}}|}_{K_{s}>>K_{p}}=\frac{1+\xi V_{s}}{1-V_{s}}$$

Combining Equations (14) and (16), we can obtain

(17) $$\xi =\frac{\tau }{1-V_{p}}$$

Finally, the transverse thermal conductivity of the systems can be estimated by Equations (15) and (17) when *V_s_* is less than $V_{s}^{c}$.

**Figure 6:**
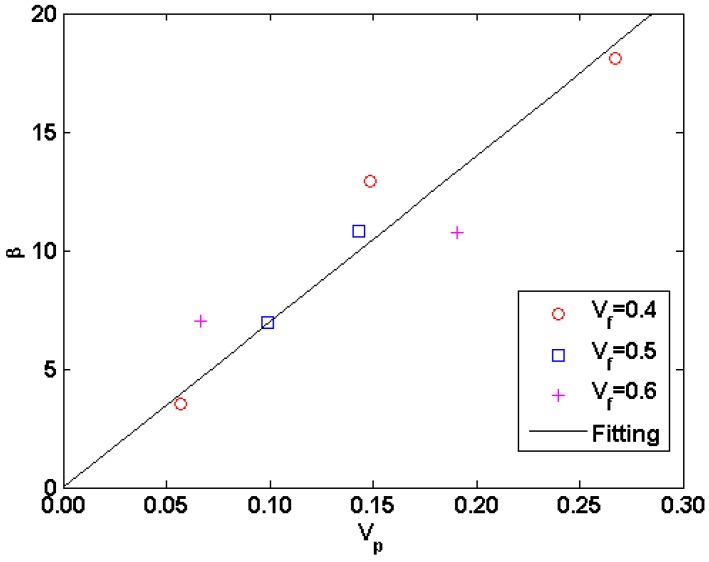
The calculated values of *β* with porosity for different two-phase systems.

When *V_s_* is larger than $V_{s}^{c}$, the pore can be seen as the isolative phase inside the solid media. Because the thermal conductivity of the pores is always less than the one of the solid by two orders, the pores can be practically seen as non-conductive. Fitting from the simulation data, 3 expression is made to put forth a porosity correction to thermal conductivity:

(18) $$\frac{K_{e}^{T}}{K_{s}}=\frac{1-V_{p}}{1+\beta V_{p}}$$

where *β* is a pore shape factor. The obtained *β* values are plotted in Figure 6. It is found that the fluctuation of *β* values is approximately increased with porosity throughout the entire porosity range. The values of *β* are estimated as *β* = 70*V_p_* with *R*^2^ ≈ 0.88. Such high level of dispersion demonstrates that the thermal conductivity of the two-phase system could considerably be affected by the randomness of the geometry of the pores.

According to the previous discussions, the complete analytical model for four-phase system (as depicted in Figure 2f) is developed as follow. The fiber coated with two layers of materials can be seen as the equivalent homogeneous solid, whose thermal conductivity is estimated by using Hasselman and Johnson’s model twice, first to replace the thermal conductivity of fiber coated with interface layer with the homogenous value, and second to replace the thermal conductivity of fiber coated with two layers of materials with the homogenous value, then the four-phase systems reduced to the two-phase systems. The complete analytical expressions are listed in Table 1.

**Figure 7:**
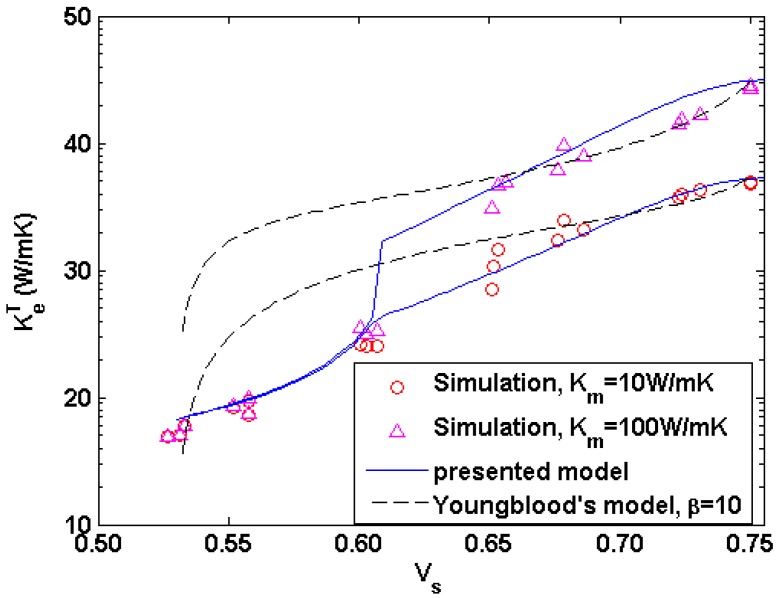
Comparison of the calculated transverse thermal conductivity of four-phase system and the predicted values by the presented and Youngblood’s expressions for $K_{f}^{T}$ = 1 W·m^−1^·K^−1^, *K_i_* = 40 W·m^−1^·K^−1^, *K_m_* = 10 and 100 W·m^−1^·K^−1^, *K_p_* = 0.01 W·m^−1^·K^−1^, *V_f_* = 0.5 and *V_i_* = 0.05.

To validate the presented model, by assuming the perfect interface conductance and setting $K_{f}^{T}$ = 1 W·m^−1^·K^−1^, *K_i_* = 40 W·m^−1^·K^−1^, *K_m_* = 10 and 100 W·m^−1^·K^−1^, *K_p_* = 0.01 W m^−1^·K^−1^, *V_f_* = 0.5 and *V_i_* = 0.05, the transverse thermal conductivity of the densified microstructure with the solid volume fraction during the CVI process are calculated and compared with the presented and Youngblood’s expressions [35] in Figure 7. In Youngblood’s model, the thermal conductivity of matrix containing pores can be estimated as the one of homogeneous media, $\bar{K_{m}}$ by the Maxwell-Eucken expression:

(19) $$\bar{K_{m}}=K_{m}\left(1-\frac{V_{p}}{V_{m}+V_{p}}\right)/\left(1+\beta \frac{V_{p}}{V_{m}+V_{p}}\right)$$

Then the effective thermal conductivity of a composite with a well-bonded fiber coating can be estimated by “3-cylinder” Markworth’s model [36]:

(20) $$K_{e}^{T}=\bar{K_{m}}C/D$$

where *C* and *D* are given by

(21) $$\begin{array}{l} C=2\frac{K_{i}}{\bar{K_{m}}}\left(\frac{K_{f}^{T}}{\bar{K_{m}}}+\frac{K_{i}}{\bar{K_{m}}}\right)\left[1+V_{f}{\left(1+\frac{r_{\text{int}}}{r_{f}}\right)}^{2}\right]\\ +\left[\left(\frac{K_{i}}{\bar{K_{m}}}-1\right)+V_{f}{\left(1+\frac{r_{\text{int}}}{r_{f}}\right)}^{2}\left(\frac{K_{i}}{\bar{K_{m}}}+1\right)\right]\left\{\frac{\frac{K_{f}^{T}}{\bar{K_{m}}}-\frac{K_{i}}{\bar{K_{m}}}}{{\left(1+\frac{r_{\text{int}}}{r_{f}}\right)}^{2}}-\left(\frac{K_{f}^{T}}{\bar{K_{m}}}+\frac{K_{i}}{\bar{K_{m}}}\right)\right\} \end{array}$$

(22) $$\begin{array}{l} D=2\frac{K_{i}}{\bar{K_{m}}}\left(\frac{K_{f}^{T}}{\bar{K_{m}}}+\frac{K_{i}}{\bar{K_{m}}}\right)\left[1-V_{f}{\left(1+\frac{r_{\text{int}}}{r_{f}}\right)}^{2}\right]\\ +\left[\left(\frac{K_{i}}{\bar{K_{m}}}-1\right)-V_{f}{\left(1+\frac{r_{\text{int}}}{r_{f}}\right)}^{2}\left(\frac{K_{i}}{\bar{K_{m}}}+1\right)\right]\left\{\frac{\frac{K_{f}^{T}}{\bar{K_{m}}}-\frac{K_{i}}{\bar{K_{m}}}}{{\left(1+\frac{r_{\text{int}}}{r_{f}}\right)}^{2}}-\left(\frac{K_{f}^{T}}{\bar{K_{m}}}+\frac{K_{i}}{\bar{K_{m}}}\right)\right\} \end{array}$$

where *r*_int_ is the thickness of the interface layer. It is clearly shown that the present model is consistent with the simulation results throughout the entire range of solid fraction, while Youngblood’s model agrees well with the simulation results by setting β value as 10 when porosity is less than 20%.

### 2.3. The Effect of Matrix Cracking on the Global Thermal Conductivity

Due to the matrix cracking and interfacial de-bonding caused by the coefficient of thermal expansion (CTE) mismatch [37] after the processing, the effective thermal conductivity values predicted by the model will be larger than the actual values in practice. In the transverse direction, the matrix cracking plays little effect on the thermal conductivity if the cracking density is not very high. Thus, the H-J model which takes the interfacial de-bonding into account is sufficient, and the effective interfacial conductance in the model is considered as an average over all fibers and a distribution of de-bonding [16]. In the longitudinal direction, Lu and Hutchinson [38] pointed out that the reduction of thermal conductivity due to matrix cracking can be as large as 50%–70% if the fiber conductivity is comparable to that of matrix and the fiber volume fraction is at the range of 0.3–0.5, and derived a model with matrix cracking density based on shear-lag analysis:

(23) $$K_{e}^{L}=K_{0}^{L}{\left[1+\frac{\left(1-V_{f}\right)K_{m}}{V_{f}K_{f}^{L}}\frac{\text{tanh}\left(\eta_{c}l_{c}/2r_{f}\right)/\left(\eta_{c}l_{c}/2r_{f}\right)}{1+\left(2K_{0}^{L}H_{c}r_{f}/\eta_{c}K_{f}^{L}V_{f}K_{m}\right)\text{tanh}\left(\eta_{c}l_{c}/2r_{f}\right)}\right]}^{-1}\;\text{with}\;\eta_{c}={\left[\frac{8K_{f}^{T}K_{0}^{L}}{\left(1-V_{f}\right)K_{f}^{L}K_{m}}\right]}^{1/2}$$

where $K_{0}^{L}$ is the initial effective conductivity, $K_{f}^{L}$ is the longitudinal thermal conductivity of the fiber, *l_c_* is the spacing between matrix cracks, and *H_c_* is the thermal conductance of the matrix cracks. The estimated microstructural quantities used in the model are listed in Table 2.

## 3. The Bundle Structures

### 3.1. Description of the Model

Recently, we used the nonconservative level set finite-element method to simulate the evolution of the boundaries of the solid phase for the complex bundle structures and calculate the gas transport properties for CVI process [21,22]. In this study, the same method was used to simulate the macro-pore structure of 2D woven and 3D braided bundle structures. The details of this method can be found in Reference [41] and are summarized briefly here. In this method the interface between solid and void phases is represented by a smooth step function of space and time, called level set function ϕ. ϕ equals zero in a domain which represents the void phase and one in the other which represents the solid phase. Across the interface, there is a smooth transition from zero to one, and it is defined by the 0.5 isocontour.

The level set method is first used to simulate the growth of the boundaries of solid phase. Then the conduction within the simulated structures is governed by the following form of Laplace’s Equation:

(24) ∇⋅[H(ϕ)∇T]=0

where *T* denotes temperature, *H* a material index which identifies the sold and void phases, and is defined as the function of ϕ:

(25) $$H\left(\phi \right)=K_{p}+\left(K_{s}-K_{p}\right)\left[\phi +\frac{\text{sin}\left(\pi \left(2\phi -1\right)\right)}{2\pi }\right]$$

Appling the corresponding temperature and thermal insulating boundaries, the effective thermal conductivity *K_e_* can be calculated [24,42].

**Figure 8:**
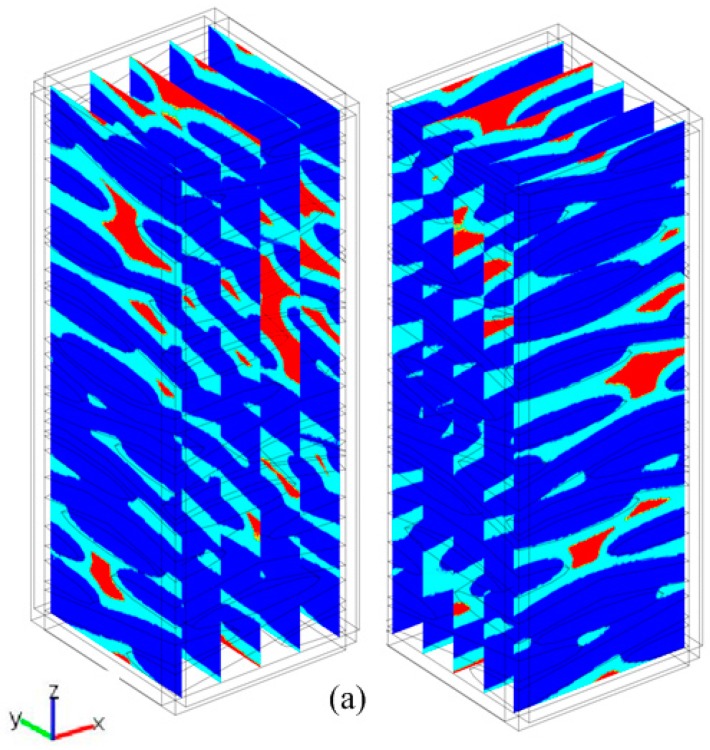

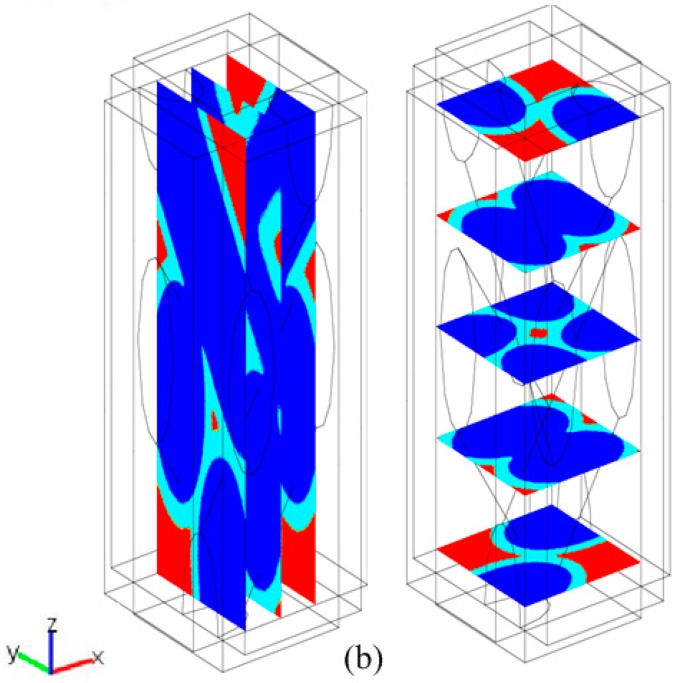
Illustration of the models for calculation of thermal conductivity of (**a**) 2D woven and (**b**) 3D braided fiber structures.

The simulated structure for 2D woven composites is shown in Figure 8a. In the constructed unit cell, we assume 2D woven composites are composed of the fiber tows with elliptical section, the tows follow a sinusoidal path and ten laminates are randomly stacked in 0°/90° sequence. By fixing the ratio of the gap width to the tow width between two adjacent tows in the longitudinal direction as 0.20 and varying the ratio of tow width to tow thickness, *e* from 5 to 10, the initial macro-porosities of the simulated structures are 0.28–0.32, which is consistent with the experimental data [43]. Thus, the simulated structures adopting these geometric parameters are considered to be appropriate for the estimation of the thermal transport properties. For convenience of illustration, the longitudinal and through-thickness directions are considered as the x and z directions, respectively. The simulated structure for 3D braided composites is shown in Figure 8b. In the constructed unit cell, Chen and Choy’s analytical model [44] is adopted and the braiding angle is setting as 20°. For convenience of illustration, the transverse and longitudinal directions are considered as the x and z directions, respectively.

### 3.2. The Thermal Conductivity Model for Bundle Structures

**Figure 9:**
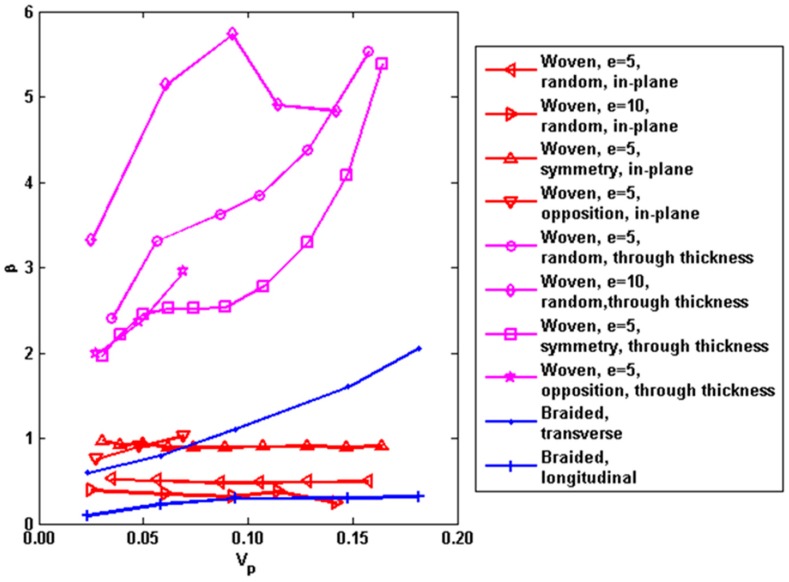
The calculated values of *β* with macro-porosity for different macro-pore structures.

Assuming bundle and matrix are isotropic and the thermal conductivity of both are equal to *K_s_,* and considering the thermal conductivity of the solid is much larger than that of void, the thermal conductivity of the composites can be expressed by Maxwell-Eucken expression:

(26) $$K_{e}=K_{s}\left(1-V_{p}\right)/\left(1+\beta V_{p}\right)$$

where *β* is a pore shape factor. The obtained *β* values by our simulation are plotted in Figure 9 and the estimated β values for different macro-pore structures are summarized in Table 3. It is found that *β* values generally increase with the average angle of the bundle orientation with respect to the global heat transfer direction, in other words, the reduction of the thermal conductivity increase with the angle of the bundle orientation with respect to the global heat transfer direction. Affected by the different geometries of the macro-pores, *β* values may vary by about 60% or less. Another interesting finding is that *β* values of the random stacking are not between the ones of the symmetry stacking and the ones of the opposition stacking. Here the symmetry stacking means all layers lie exactly one over each other, while the opposition stacking means the layer is shifted along the in-plane direction to make the raised portion of the layer insert into the hole of the adjacent one. This foundation means the thermal conductivity of the random stacking is not between the one of the symmetry stacking and the one of the opposition stacking at the same macro-porosity, which is opposite of the acknowledgement for gas transport of 2D woven composites [45].

In Table 4 and Table 5, we test the sensitivity of the model to *β* values. The results show that the dispersion of *β* values has a small effect, order of 10% or less, on the thermal conductivities of 2D woven composite along the in-plane direction and the ones of 3D braided composites in the longitudinal direction, while the thermal conductivities of 2D woven composite along the through-thickness direction and the ones of 3D braided composites in the transverse direction vary by about 30% and 15% with *β* values, respectively. The residual macro-porosity is about 10% for the composites densified by CVI, then the uncertainty in the thermal conductivities in the transverse direction due to the macro-pore geometry is on the order of ~10%.

In the actual case, the thermal conductivity of bundle is not equal to that of matrix, the bundle and matrix may show different heat transfer capabilities in the transverse and axial directions. Hence our model should be revised to consider this heterogeneity. Considering the matrix are deposited on the surface of the bundles, then it generally follows the same direction of the bundle, hence the average thermal conductivity of the solid phase can also be approximated by the function related to the average angle of the bundle orientation with respect to the global heat transfer direction, θ:

(27) $$K_{s}=\left(K_{b}^{\left|\right|}\frac{V_{b}}{V_{s}}+K_{m}\frac{V_{m}}{V_{s}}\right){\text{cos}}^{2}\;\theta +{\left[\frac{V_{b}/V_{s}}{K_{b}^{⊥}}+\frac{V_{m}/V_{s}}{K_{m}}\right]}^{-1}{\text{sin}}^{2}\;\theta$$

where $K_{b}^{\left|\right|}$, $K_{b}^{⊥}$ are the thermal conductivities of the bundle in the axial and transverse direction, respectively. For 2D woven composites along the in-plane direction, only a half of the total bundles is parallel to the heat transfer direction, while the other half is transverse to the heat transfer direction, thus θ = 45°. For 2D woven composites along the through-thickness direction, the reported mean crimp angles are between 1° and 5°, thus we choose 2° in this study and θ = 90° − 2° = 88°. For 3D woven composites in the longitudinal direction, the bundles are laid along the transfer direction axis at a small braiding angle (~20°), and thus θ = 20°. For 3D woven composites in the transverse direction, a half of the total bundles is laid along the heat transfer direction axis at an angle of (90° − 20°), while the other half is totally transverse to the heat transfer direction, thus θ = (90° + 70°)/2 = 80°.

**Figure 10:**
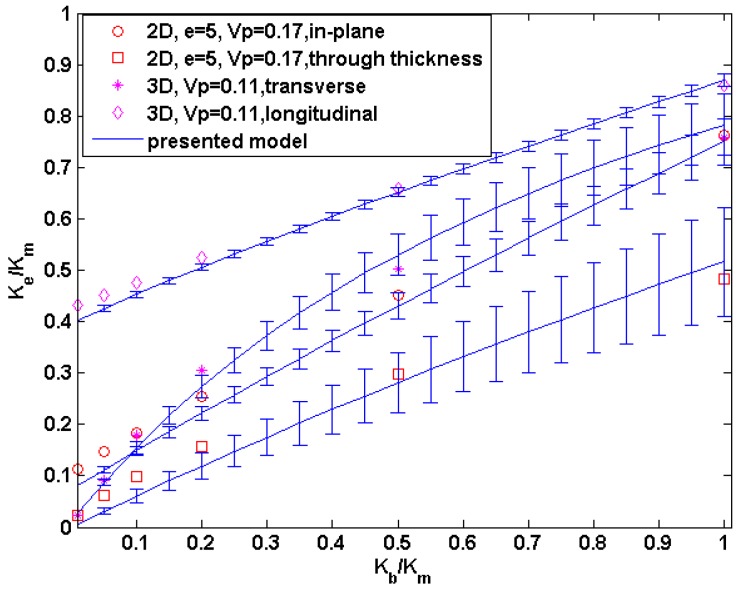
The reduced thermal conductivity of the three-phase bundle structures with *K_p_*/*K_s_* = 0.01. The bundles and the matrix are considered as isotropic and the bundles thickness is 140 μm. The error bars reflect the range of *K_e_* due to the uncertainty of *β* values.

Combining Equations (26) and (27), we can estimate the thermal conductivity of CVI-densified composites with anisotropic bundle and matrix at the different stage of densification. Because of the difficulties of determining the local conductivity orientation in the numerical mode, the model is only validated by our simulation by considering the bundles and the matrix as isotropic, and the comparison with the predictions of our model and numerical results are shown in Figure 10. It is found that the model agrees well with the simulation results when *K_b_*/*K_m_* is larger than 0.5, and slightly under-estimates the thermal conductivity when *K_b_*/*K_m_* is less than 0.5.

As discussed in the Section 2, the matrix cracking may play an important role in the parallel direction of thermal transport. Then Lu and Hutchinson’s model [38] (Equation (23)) should be adopted to estimate the reduction of thermal conductivity of the macro-pore structures, thus Equation (27) could be revised as:

(28) $$K_{s}=K_{s}^{L}{\text{cos}}^{2}\;\theta +{\left[\frac{V_{b}/V_{s}}{K_{b}^{⊥}}+\frac{V_{m}/V_{s}}{K_{m}}\right]}^{-1}{\text{sin}}^{2}\;\theta$$

with $K_{s}^{L}=K_{0}^{L}{\left[1+\frac{\left(1-\left(V_{b}/V_{s}\right)\right)K_{m}}{V_{b}K_{b}^{\left|\right|}/V_{s}}\frac{\text{tanh}\left(\eta_{c}l_{c}/2r_{b}\right)/\left(\eta_{c}l_{c}/2r_{b}\right)}{1+\left(2r_{b}V_{s}K_{0}^{L}H_{c}/V_{b}\eta_{c}K_{b}^{\left|\right|}K_{m}\right)\text{tanh}\left(\eta_{c}l_{c}/2r_{b}\right)}\right]}^{-1}$, $K_{0}^{L}=K_{b}^{\left|\right|}\frac{V_{b}}{V_{s}}+K_{m}\frac{V_{m}}{V_{s}}$ and $\eta_{c}={\left[\frac{8K_{b}^{⊥}K_{0}^{L}}{\left(1-\left(V_{b}/V_{s}\right)\right)K_{b}^{\left|\right|}K_{m}}\right]}^{1/2}$ where $r_{b}$ is the equivalent radius of the bundle and can be seen as the geometric mean of the half length of the major and minor axis of the bundle cross-section. For the ease of estimation, the values of *l_c_* and *H_c_* listed in Table 2 are also used for macro-pore structures.

## 4. Sensitive Analysis of Components’ Parameters

In the previous sections, the models which relate the thermal conductivities of the CVI-densified composites with their constituent properties have been presented. However, the reliable estimation of thermal conductivity of these composites also depends on the precise thermal data and volume fractions of their components. However, most of the time these parameters may be not precisely known. Thus, before comparing with our models and the experimental data, the sensitivities of the predicted thermal conductivities to these parameters are assessed.

The adopted thermal conductivities and volume fractions of the components are determined first and listed in Table 6 and Table 7, respectively, which are obtained from the reliable sources or by the reasonable estimations. For instance, Taylor et al.’s experimental data [46] exhibits the axial thermal conductivity of T-300 carbon fiber would not changed after any heat treatment, if the temperature of the heat treatment is below 1400 K. Since the material are usually processed at around 1300 K, the thermal conductivity of T-300 carbon fiber at room temperature, 8 W·m^−1^·K^−1^ is safely retained [47]. According to Jumel et al.’s measurements [48], pan-based carbon fiber is transverse isotropic, the ratio of the axial thermal conductivity to the transverse one is 10.5 after any heat treatment, hence the transverse thermal conductivity of T-300 fiber is considered to be about 0.8 W·m^−1^·K^−1^ in this study. PyC interface layer displays a cylindrical symmetry in the transverse direction, hence the average values estimated by Katoh et al. [49] and Youngblood et al. [16] for the PyC interface layer have been retained. The estimated thermal data of CVI SiC matrix by Yamada et al. [50] is adopted. For the ease of the estimation, the spacing and thermal conductance of micro-cracks and macro-cracks are assumed to be equal and listed in Table 2. By inputting the listed data, changing only one parameter and fixing others, a detailed sensitivity study to model’s parameters has been performed.

We had performed the sensitive analysis for 1D and 2D composites reinforced by two fibers. It can be found that composites’ thermal conductivities are not very sensitive to components’ parameters, composites’ thermal conductivities vary with these parameters only by less than 10%. The uncertainties of these parameters do not destroy the robustness of the model, their values can be utilized safely. The only exceptions are *V_p_^mic^*, *V_p_^mac^*, *V_b_* and *K_m_*, as shown by Table 8, Table 9, Table 10, Table 11 and Table 12. These results demonstrate the uncertainties of these parameters play a significant influence on the composites’ thermal conductivities, the fluctuation of the calculated thermal conductivities will be on the order of about 20%. Another foundation is the variation of composites’ thermal conductivities will almost linearly vary with the relative difference of these parameters, since the reduced sensitivity index (RSI) do not vary very much with the relative difference of these parameters.

**Figure 11:**
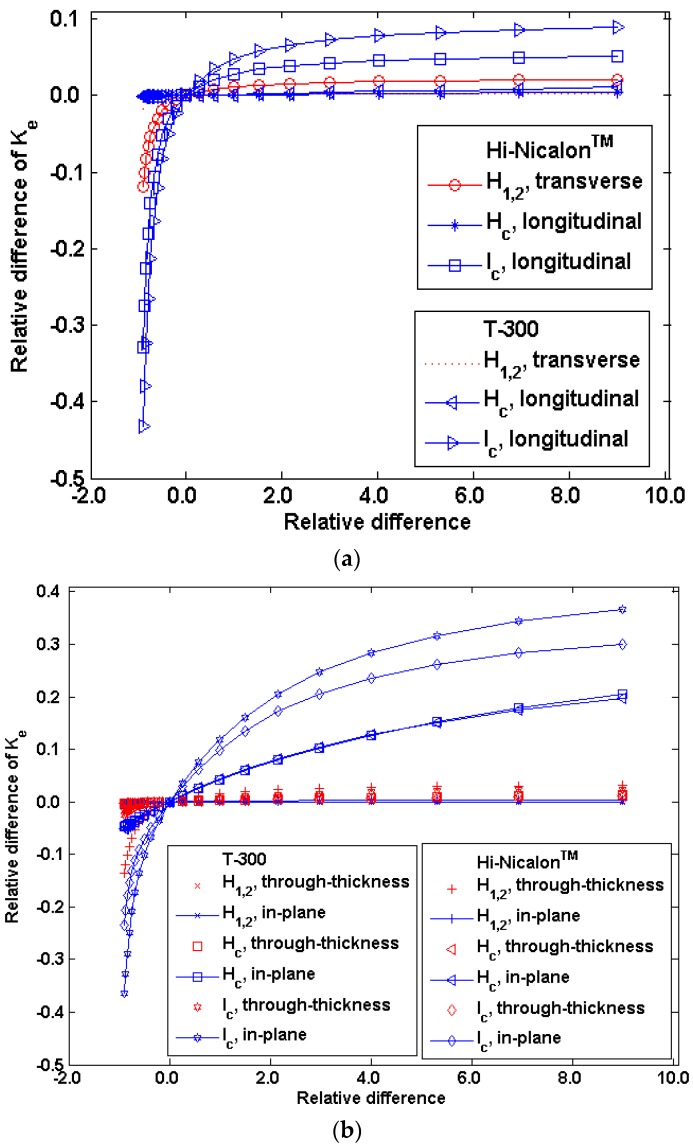
The sensitivity of theeffective thermal conductivity to the matrix cracking parameters *H*_1,2_, *H_c_* and *l_c_*: (**a**) 1D and (**b**) 2D composites.

Except for the above parameters, some other parameters (e.g., the parameters of the matrix cracking) may vary in a much larger range, due to the uncontrollability of the processing. Hence, the sensitivity analysis in a larger range for these parameters are performed and shown in Figure 11. The results in Figure 11 demonstrate that the spacing between matrix cracks *l_c_* plays the most influence on the composites’ thermal conductivities. Reducing *l_c_* by 90% will cause the reduction of composites’ thermal conductivities by 30%–40%. However, increasing *l_c_* by one order will cause the increases of composites’ thermal conductivities by 5%–10% and 20%–40% for 1D and 2D composites, respectively. These results demonstrate that the variance and contradiction of the thermal conductivity-residual porosity relation between different reports may partly due to the difficulty to distinguish the residual micro-porosity and macro-porosity, since the contributions of both to the thermal conductivity differ greatly. The second reason is the ignorance of the influence of the matrix cracking on the matrix thermal conductivity.

## 5. Comparison with Experimental Data

In Table 13, we summarize the available experimental data with the explicit geometrical information of various CVI-densified composites from various resources [13,15,16,35,53,54], adopt the thermal data of components listed in Table 6 and estimate the thermal conductivity of CVI-densified SiC matrix by our model. For the ease of estimation, the volume fraction of bundle and the volume fraction of the residual micro-porosity inside the bundle are utilizing the listed values in Table 7. It is noted that the measured 2D woven composites in Reference [13] stacked in 0°/30°/60°, which is slightly different from our geometrical model. We assume the difference of the stacking sequence on the thermal transport properties is not very large, and consider our model is still available. It is found that the estimated values by the present models are well consistent with the measured values. According to the discussion in Section 4, the departures may mainly attribute to the uncertainties of residual porosity in transverse direction and of the matrix cracking’s density.

## 6. Conclusions

The numerical computations of the thermal conductivities of unidirectional, 2D woven and 3D braided composites at various stages of CVI densification are performed. The effects of the complex dual-scale pore structures on the thermal transport properties have been studied and two analytical thermal conductivity models with some empirical coefficients have been developed.

In general, there are seven adjustable parameters are uncertain or not available through experimental measurements and reported literatures:

(i) Micro-porosity inside the bundle for the 2D and 3D composites, *V_p_^mac^*
(ii) Macro-porosity between the bundle for the 2D and 3D composites, *V_p_^mac^*
(iii) Volume fraction of the bundle for the 2D and 3D composites, *V_b_*
(iv) Pore shape factor for the 2D and 3D composites, *β*
(v) Effective fiber-interface and interface-matrix interfacial conductance, *H*_1__,2_
(vi) Thermal conductance of the matrix cracks, *H_c_*
(vii) Spacing between matrix cracks, *l_c_*

Through these parameters, the effective thermal conductivity of the CVi-densified composites have been found to be the most sensitive to the spacing between matrix cracks or matrix cracking’s density, followed by volume fraction of the bundle and thermal conductance of the matrix cracks, finally by micro-porosity inside the bundles and macro-porosity between the bundles.

Although our model is developed in a simplified geometry, while the structures of actual composites are certainly more complex than these idealized structures, the presented models can provide insights into the magnitude of composites’ thermal conductivities, provide an effective tool to assess the effects of all parameters on composites’ thermal conductivities, and are useful for designing material processing and performance.

## Figures and Tables

**Table 1:** The analytical model for transverse thermal conductivity of unidirectional composites during the CVI process.

Expression
When $V_{s}\leq V_{s}^{c}$ $K_{e}^{T}=K_{p}\left(1+\xi \eta V_{s}\right)/\left(1-\eta V_{s}\right)$ When $V_{s}>V_{s}^{c}$ $K_{e}^{T}=K_{fim}\left(1-V_{p}\right)/\left(1+\beta V_{p}\right)$ with $\eta =\left(K_{fim}/K_{p}-1\right)/\left(K_{fim}/K_{p}+\xi \right)$, $\xi =\frac{\tau }{1-V_{p}}$ ^†^, $V_{s}^{c}=1-0.33{\left(1-V_{f}\right)}^{0.19}$, $K_{fim}=K_{m}\frac{\left(\frac{K_{fi}^{T}}{K_{m}}-1-\frac{K_{fi}^{T}}{r_{fi}H_{2}}\right)\left(\frac{V_{f}+V_{i}}{V_{s}}\right)+\left(1+\frac{K_{fi}^{T}}{r_{fi}H_{2}}+\frac{K_{fi}^{T}}{K_{m}}\right)}{\left(1+\frac{K_{fi}^{T}}{r_{fi}H_{2}}-\frac{K_{fi}^{T}}{K_{m}}\right)\left(\frac{V_{f}+V_{i}}{V_{s}}\right)+\left(1+\frac{K_{fi}^{T}}{r_{fi}H_{2}}+\frac{K_{fi}^{T}}{K_{m}}\right)}$ ^‡^ and $K_{fi}^{T}=K_{i}\frac{\left(\frac{K_{f}^{T}}{K_{i}}-1-\frac{K_{f}^{T}}{r_{f}H_{1}}\right)\left(\frac{V_{f}}{V_{f}+V_{i}}\right)+\left(1+\frac{K_{f}^{T}}{r_{f}H_{1}}+\frac{K_{f}^{T}}{K_{i}}\right)}{\left(1+\frac{K_{f}^{T}}{r_{f}H_{1}}-\frac{K_{f}^{T}}{K_{i}}\right)\left(\frac{V_{f}}{V_{f}+V_{i}}\right)+\left(1+\frac{K_{f}^{T}}{r_{f}H_{1}}+\frac{K_{f}^{T}}{K_{i}}\right)}$ ^‡^

^†^ When *V_f_* = 50%, $\tau =0.72\times {\left(\frac{1-0.29}{V_{p}-0.29}\right)}^{0.82}$, The estimation of τ for other *V_f_* values can refer to Table II of Reference [22]. ^‡^ *H*_1_ and *H*_2_ denote the effective fiber-interface and interface-matrix interfacial conductance, respectively.

**Table 2:** Microstructural data for the model.

Parameter	Value	Source
*H*_1,2_ (W/m^2^K)	2.4 × 10^7^	[16]
*H_c_* (W/m^2^K)	1.6 × 10^4^	[39]
*l_c_* (mm)	0.15	[40]

**Table 3:** The calculated β values for different macro-pore structures.

1D, Transverse	2D, In-Plane	2D, Through Thickness	3D, Longitudinal	3D, Transverse
21 ± 17	0.62 ± 0.38	3.9 ± 2.0	0.21 ± 0.12	1.3 ± 0.8

**Table 4:** Sensitivity analysis of the pore shape factor for 2D woven and 3D braided composites.

2D, in-Plane	RD of *β* (%)	−61.3	−36.8	−18.4	18.4	36.8	61.3
*V_p_* = 20%	RD of *K_e_* (%)	7.3	4.2	2.1	2.0	3.9	6.3
	RSI (%)	−11.8	−11.5	−11.3	−10.8	−10.6	−10.3
*V_p_* = 15%	RD of *K_e_* (%)	5.5	3.2	1.6	−1.5	−3.0	−5.0
	RSI (%)	−9.0	−8.8	−8.6	−8.4	−8.2	−8.1
*V_p_* = 10%	RD of *K_e_* (%)	3.7	2.2	1.1	−1.1	−2.1	−3.5
	RSI (%)	−6.1	−6.0	−5.9	−5.8	−5.7	−5.6
*V_p_* = 5%	RD of *K_e_* (%)	1.9	1.1	0.6	−0.5	−1.1	−1.8
	RSI (%)	−3.1	−3.0	−3.0	−3.0	−3.0	−3.0
**2D, Through Thickness**	**RD of *β* (%)**	**−51.3**	**−35.9**	**−20.5**	**20.5**	**35.9**	**51.3**
*V_p_* = 20%	RD of *K_e_* (%)	29.0	18.7	9.9	−8.2	−13.6	−18.3
	RSI (%)	−56.5	−52.0	−48.2	−40.2	−37.9	−35.8
*V_p_* = 15%	RD of *K_e_* (%)	23.3	15.3	8.2	−7.0	−11.7	−15.9
	RSI (%)	−45.5	−42.5	−40.0	−34.3	−32.6	−31.0
*V_p_* = 10%	RD of *K_e_* (%)	16.8	11.2	6.1	−5.4	−9.2	−12.6
	RSI (%)	−32.8	−31.2	−29.8	−26.5	−25.5	−24.5
*V_p_* = 5%	RD of *K_e_* (%)	9.1	6.2	3.5	−3.2	−5.5	−7.7
	RSI (%)	−17.8	−17.3	−16.9	−15.8	−15.4	−15.1

Relative difference (RD) of *β* = (*β*-*β*^Ref^)/*β*^Ref^; Relative difference (RD) of *K_e_* = (*K_e_*-*K_e_*^Ref^)/*K_e_*^Ref^; Reduced sensitivity index (RSI) = [(*K_e_*-*K_e_*^Ref^)/*K_e_*^Ref^]/[(*β*-*β*^Ref^)/*β*^Ref^].

**Table 5:** Sensitivity analysis of the pore shape factor for 3D braided composites.

3D, Longitudinal	RD of *β* (%)	−61.5	−43.1	−18.5	18.5	43.1	61.5
*V_p_* = 20%	RD of *K_e_* (%)	14.5	9.8	4.0	−3.7	−8.2	−11.3
	RSI	−0.237	−0.226	−0.214	−0.198	−0.189	−0.183
*V_p_* = 15%	RD of *K_e_* (%)	11.2	7.6	3.1	−2.9	−6.6	−9.1
	RSI	−0.182	−0.175	−0.168	−0.158	−0.152	−0.148
*V_p_* = 10%	RD of *K_e_* (%)	7.6	5.2	2.2	−2.1	−4.7	−6.6
	RSI	−0.124	−0.121	−0.117	−0.112	−0.110	−0.108
*V_p_* = 5%	RD of *K_e_* (%)	3.9	2.7	1.1	−1.1	−2.6	−3.6
	RSI	−0.063	−0.063	−0.062	−0.060	−0.059	−0.059
**3D, Transverse**	**RD of *β* (%)**	**−57.1**	**−40.0**	**−17.1**	**17.1**	**40.0**	**57.1**
*V_p_* = 20%	RD of *K_e_* (%)	2.4	1.6	0.7	−0.7	−1.6	−2.3
	RSI	−0.041	−0.041	−0.041	−0.040	−0.040	−0.039
*V_p_* = 15%	RD of *K_e_* (%)	1.8	1.2	0.5	−0.5	−1.2	−1.7
	RSI	−0.031	−0.031	−0.031	−0.030	−0.030	−0.030
*V_p_* = 10%	RD of *K_e_* (%)	1.2	0.8	0.4	−0.4	−0.8	−1.2
	RSI	−0.021	−0.021	−0.021	−0.021	−0.020	−0.020
*V_p_* = 5%	RD of *K_e_* (%)	0.6	0.4	0.2	−0.2	−0.4	−0.6
	RSI	−0.010	−0.010	−0.010	−0.010	−0.010	−0.010

**Table 6:** Reported data of the components.

Material	Thermal Conductivity (W/m/K)	Fiber Radius (μm)	Half of Bundle Thickness (μm)	Source
Nicalon	2.2	7	~140	[51]
Hi-Nicalon™	6.4	7	~140	[51]
Tyrrano SA	60	~4	~140	[50]
T-300, Transverse	0.8	3.5	~100	[46,47]
T-300, Axial	8	3.5	-	[52]
PyC, Transverse	24.5	-	-	[9,16,49]
PyC, Axial	114	-	-	[9]
SiC Matrix	67	-	-	[50]
T-300, Transverse	0.8	3.5	~100	[46,47]
T-300, Axial	8	3.5	-	[52]
PyC, Transverse	24.5	-	-	[9,16,49]
PyC, Axial	114	-	-	[9]
SiC Matrix	67	-	-	[50]

**Table 7:** Estimated volume fractions of the components for 1D, 2D and 3D composites.

Parameter	Value
Volume fraction of fiber in 1D composites or in the bundles of 2D and 3D composites, *V_f_^1D^*	55%
Volume fraction of PyC interface layer in 1D composites or in the bundles of 2D and 3D composites, *V_i_^1D^*	5%
Volume fraction of micro-pores in 1D composites or in the bundles of 2D and 3D composites *V_p_^mic^*	8%
Volume fraction of bundles in 2D and 3D composites *V_b_*	73%
Volume fraction of macro-pores in 2D and 3D composites *V_p_^mac^*	5%

**Table 8:** Sensitivity analysis of porosity for 1D composites.

Direction	Fiber	RD of *V_p_^mic^* (%)	−20.0	−10.0	10.0	20.0
Longitudinal	Hi-Nicalon™	RD of *K_e_* (%)	19.9	10.1	−10.3	−20.9
		RSI	−0.994	−1.01	−1.03	−1.04
Transverse	-	RD of *K_e_* (%)	29.8	14.4	−13.5	−26.2
		RSI	−1.49	−1.44	−1.35	−1.31
Longitudinal	T-300	RD of *K_e_* (%)	15.6	8.2	−8.7	−17.9
		RSI	−0.780	−0.816	−0.873	−0.896
Transverse	-	RD of *K_e_* (%)	40.7	19.6	−18.2	−35.2
		RSI	−2.04	−1.96	−1.82	−1.76

**Table 9:** Sensitivity analysis of thermal conductivity of matrix for 1D composites.

Direction	Fiber	RD of *K_m_* (%)	−20.0	−10.0	10.0	20.0
Longitudinal	Hi-Nicalon™	RD of *K_e_* (%)	−13.5	−6.8	6.7	13.5
		RSI	0.675	0.675	0.674	0.675
Transverse	-	RD of *K_e_* (%)	−13.9	−7.0	6.9	0.139
		RSI	0.696	0.695	0.693	0.693
Longitudinal	T-300	RD of *K_e_* (%)	−12.2	−6.1	6.1	12.2
		RSI	0.609	0.609	0.609	0.609
Transverse	-	RD of *K_e_* (%)	−17.8	−8.9	8.9	17.8
		RSI	0.891	0.891	0.890	0.891

**Table 10:** Sensitivity analysis of macro-porosity for 2D composites.

Direction	Fiber	RD of *V_p_^mac^* (%)	−50.0	−25.0	25.0	50.0
Longitudinal	Hi-Nicalon™	RD of *K_e_* (%)	3.9	2.0	−1.9	−3.8
		RSI	−0.079	−0.078	−0.076	−0.076
Transverse	-	RD of *K_e_* (%)	13.8	6.6	−6.1	−11.7
		RSI	−0.277	−0.264	−0.243	−0.234
Longitudinal	T-300	RD of *K_e_* (%)	4.0	2.0	−1.9	−3.8
		RSI	−0.079	−0.079	−0.077	−0.077
Transverse	-	RD of *K_e_* (%)	14.0	6.7	−6.1	−11.8
		RSI	−0.279	−0.267	−0.245	−0.235

**Table 11:** Sensitivity analysis of Bundle volume fraction for 2D composites.

Direction	Fiber	RD of *V_b_* (%)	−20.0	−10.0	10.0	20.0
Longitudinal	Hi-Nicalon™	RD of *K_e_* (%)	−1.8	−0.9	1.2	2.6
		RSI	0.088	0.092	0.115	0.129
Transverse	-	RD of *K_e_* (%)	16.9	7.7	−6.6	−12.3
		RSI	−0.843	−0.773	−0.661	−0.617
Longitudinal	T-300	RD of *K_e_* (%)	−1.6	−0.8	1.1	2.6
		RSI	0.082	0.082	0.106	0.128
Transverse	-	RD of *K_e_* (%)	18.1	8.2	−7.0	−12.9
		RSI	−0.903	−0.823	−0.697	−0.646

**Table 12:** Sensitivity analysis of thermal conductivity of matrix for 2D composites.

Direction	Fiber	RD of *K_m_* (%)	−20.0	−10.0	10.0	20.0
Longitudinal	Hi-Nicalon™	RD of *K_e_* (%)	−10.9	−5.4	5.5	10.9
		RSI	0.543	0.543	0.545	0.546
Transverse	-	RD of *K_e_* (%)	−13.1	−6.5	6.5	12.9
		RSI	0.656	0.652	0.646	0.644
Longitudinal	T-300	RD of *K_e_* (%)	−10.1	−5.1	5.1	10.2
		RSI	0.506	0.507	0.508	0.509
Transverse	-	RD of *K_e_* (%)	−16.7	−8.3	8.3	16.5
		RSI	0.836	0.833	0.827	0.824

**Table 13:** Measured thermal conductivities and calculated values of CVI-deposited composites.

Reference	Fiber	*V_p_* (%)	*V_f_* (%)	*V_i_* (%)	*V_m_* (%)	$K_{e}^{\text{exp}}$ (W/m/K)	$K_{e}^{pred}$ (W/m/K)
2D Woven Composites, through Thickness							
[13,53]	Nicalon	13.7	38.9	0.3	47.1	6.2	5.9
		18.7	35.7	9.0	36.6	8.7	8.7
		14.0	40	4.4	41.6	4.6	4.6
2D Woven Composites, in-Plane							
[54]	T-300	5.9	40.2	4.5	49.4	16.9	16.2
3D braided Composites, Longitudinal							
[15,53]	T-300	7.70	40	1.46	50.84	23.0	21.7
		7.19	40	1.95	50.86	20.7	22.0

## References

[1] Besmann T.M., Sheldon B.W., Lowden R.A., Stinton D.P. (1991). Vapor-phase fabrication and properties of continuous-filament ceramic composites. Science.

[2] Golecki I. (1997). Rapid vapor-phase densification of refractory composites. Mater. Sci. Eng. R Rep..

[3] Naslain R., Langlais F., Vignoles G., Pailler R., Tandon R., Wereszczak A., Lara-Curzio E. (2008). The CVI-process: State of the art and perspective. Mechanical Properties and Performance of Engineering Ceramics II: Ceramic Engineering and Science Proceedings.

[4] Yan X.-T., Xu Y.-D. (2010). Chemical vapour infiltration. Chemical Vapour Deposition: An Integrated Engineering Design for Advanced Materials.

[5] Vignoles G.L., Boisse P. (2015). Chemical vapor deposition/infiltration processes for ceramic composites. Advances in Composites Manufacturing and Process Design.

[6] Vaidyaraman S., Lackey W.J., Agrawal P.K., Freeman G.B. (1995). Forced flow-thermal gradient chemical vapor infiltration (FCVI) for fabrication of carbon/carbon. Carbon.

[7] Tomadakis M.M., Sotirchos S.V. (1993). Effective diffusivities and conductivities of random dispersions of nonoverlapping and partially overlapping unidirectional fibers. J. Chem. Phys..

[8] Skamser D.J., Bentz D.P., Coverdale R.T., Spotz M.S., Martys N., Jennings H., Johnson D.L. (1994). Calculation of the thermal conductivity and gas permeability in a uniaxial bundle of fibers. J. Am. Ceram. Soc..

[9] Vignoles G.L., Coindreau O., Ahmadi A., Bernard D. (2007). Assessment of geometrical and transport properties of a fibrous C/C composite preform as digitized by X-ray computerized microtomography: Part II. Heat and gas transport properties. J. Mater. Res..

[10] Vignoles G.L., Boisse P. (2015). Modeling of chemical vapor infiltration processes. Advances in Composites Manufacturing and Process Design.

[11] Tomadakis M.M., Sotirchos S.V. (1996). Transport through random arrays of conductive cylinders dispersed in a conductive matrix. J. Chem. Phys..

[12] Tawil H., Bentsen L., Baskaran S., Hasselman D. (1985). Thermal diffusivity of chemically vapour deposited silicon carbide reinforced with silicon carbide or carbon fibres. J. Mater. Sci..

[13] Beecher S., Dinwiddie R., Lowden R. (1993). The thermal conductivity of carbon coated silicon carbide fibers embedded in a silicon carbide matrix. Therm. Conduct..

[14] Luo R., Liu T., Li J., Zhang H., Chen Z., Tian G. (2004). Thermophysical properties of carbon/carbon composites and physical mechanism of thermal expansion and thermal conductivity. Carbon.

[15] Cheng L., Xu Y., Zhang Q., Zhang L. (2003). Thermal diffusivity of 3D C/SiC composites from room temperature to 1400 °C. Carbon.

[16] Youngblood G.E., Senor D.J., Jones R.H., Kowbel W. (2002). Optimizing the transverse thermal conductivity of 2D-SiC_f_/SiC composites, II. Experimental. J. Nucl. Mater..

[17] Hasselman D.P.H., Venkateswaran A., Yu M., Tawil H. (1991). Role of interfacial debonding and matrix cracking in the effective thermal diffusivity of SiC fibre-reinforced chemical vapour deposited sic matrix composites. J. Mater. Sci. Lett..

[18] Whittaker A.J., Taylor R. (1990). Thermal transport properties of carbon-carbon fibre composites III. Mathematical modelling. Proc. R. Soc. Lond. Ser. A Math. Phys. Sci..

[19] Youngblood G.E., Senor D.J., Jones R.H. (2002). Optimizing the transverse thermal conductivity of 2D-SiC_f_/SiC composites. I. Modeling. J. Nucl. Mater..

[20] Youngblood G.E., Senor D.J., Jones R.H., Graham S. (2002). The transverse thermal conductivity of 2D-SiC_f_/SiC composites. Compos. Sci. Technol..

[21] Guan K., Cheng L., Zeng Q., Li H., Liu S., Li J., Zhang L. (2013). Prediction of permeability for chemical vapor infiltration. J. Am. Ceram. Soc..

[22] Guan K., Cheng L., Zeng Q., Zhang L., Deng J., Li K., Li H. (2013). Modeling of pore structure evolution between bundles of plain woven fabrics during chemical vapor infiltration process: The influence of preform geometry. J. Am. Ceram. Soc..

[23] Spitzig W.A., Kelly J.F., Richmond O. (1985). Quantitative characterization of second-phase populations. Metallography.

[24] Floury J., Carson J., Pham Q. (2008). Modelling thermal conductivity in heterogeneous media with the finite element method. Food Bioprocess. Technol..

[25] Rayleigh L. (1892). On the influence of obstacles arranged in rectangular order upon the properties of a medium. Philos. Mag.Ser. 5.

[26] Farmer J.D., Covert E.E. (1994). Transverse thermal conductance of thermosetting composite materials during their cure. J. Thermophys. Heat Transf..

[27] Wetherhold R.C., Wang J. (1994). Difficulties in the theories for predicting transverse thermal conductivity of continuous fiber composites. J. Compos. Mater..

[28] Rolfes R., Hammerschmidt U. (1995). Transverse thermal conductivity of CFRP laminates: A numerical and experimental validation of approximation formulae. Compos. Sci. Technol..

[29] Hasselman D.P.H., Johnson L.F. (1987). Effective thermal conductivity of composites with interfacial thermal barrier resistance. J. Compos. Mater..

[30] Hasselman D.P.H., Donaldson K.Y., Thomas J.R. (1993). Effective thermal conductivity of uniaxial composite with cylindrically orthotropic carbon fibers and interfacial thermal barrier. J. Compos. Mater..

[31] Keller J.B. (1964). A theorem on the conductivity of a composite medium. J. Math. Phys..

[32] Tomadakis M.M., Sotirchos S.V. (1993). Transport properties of random arrays of freely overlapping cylinders with various orientation distributions. J. Chem. Phys..

[33] Progelhof R.C., Throne J.L., Ruetsch R.R. (1976). Methods for predicting the thermal conductivity of composite systems: A review. Polym. Eng. Sci..

[34] Pilling M.W., Yates B., Black M.A., Tattersall P. (1979). The thermal conductivity of carbon fibre-reinforced composites. J. Mater. Sci..

[35] Youngblood G.E., Senor D.J., Jones R.H. (2004). Modeling the transverse thermal conductivity of 2-D SiC_f_/SiC composites made with woven fabric. Fusion Sci. Technol..

[36] Markworth A.J. (1993). The transverse thermal conductivity of a unidirectional fibre composite with fibre-matrix debonding: A calculation based on effective-medium theory. J. Mater. Sci. Lett..

[37] Hasselman D.P.H., Venkateswaran A., Tawil H. (1991). Role of interfacial debonding and matrix cracking in the effective thermal diffusivity of alumina-fiber-reinforced chemical-vapor-infiltrated silicon carbide matrix composites. J. Am. Ceram. Soc..

[38] Lu T.J., Hutchinson J.W., Rodel D.J. (1995). Effect of matrix cracking on the overall thermal conductivity of fibre-reinforced composites (and discussion). Philos. Trans. R. Soc. Lond. Ser. A Phys. Eng. Sci..

[39] Graham S., McDowell D., Lara-Curzio E., Dinwiddie R., Wang H., Jenkins M.G., Lara-Curzio E., Gonczy S.T. (2000). The effects of microstructural damage on the thermal diffusivity of continuous fiber-reinforced ceramic matrix composites. Mechanical, Thermal and Environmental Testing and Performance of Ceramic Composites and Components.

[40] Liu S., Zhang L., Yin X., Liu Y., Cheng L. (2014). Proportional limit stress and residual thermal stress of 3D SiC/SiC composite. J. Mater. Sci. Technol..

[41] Guan K., Cheng L., Zeng Q., Feng Z.-Q., Zhang L., Li H., Ren H. (2011). Modeling of pore structure evolution within the fiber bundle during chemical vapor infiltration process. Chem. Eng. Sci..

[42] Carson J.K., Lovatt S.J., Tanner D.J., Cleland A.C. (2003). An analysis of the influence of material structure on the effective thermal conductivity of theoretical porous materials using finite element simulations. Int. J. Refrig..

[43] Gélébart L., Chateau C., Bornert M., Crépin J., Boller E. (2010). X-ray tomographic characterization of the macroscopic porosity of chemical vapor infiltration SiC/SiC composites: Effects on the elastic behavior. Int. J. Appl. Ceram. Technol..

[44] Chen L., Tao X.M., Choy C.L. (1999). On the microstructure of three-dimensional braided preforms. Compos. Sci. Technol..

[45] Vignoles G.L. (1995). Modelling binary, knudsen and transition regime diffusion inside complex porous media. J. Phys. IV Colloq..

[46] Taylor R., Turner S.P., Garner K., Jiang X.X. (1993). Thermal conductivity of carbon fibres. High Temp. High Press..

[47] Yamane T., Katayama S., Todoki M., Hatta I. (2000). The measurements of thermal conductivity of carbon fibers. J. Wide Bandgap Mater..

[48] Jumel J., Lepoutre F., Roger J.-P., Neuer G., Cataldi M., Enguehart F. (2003). Microscopic thermal characterization of composites. Rev. Sci. Instrum..

[49] Katoh Y., Nozawa T., Snead L.L., Hinoki T., Kohyama A. (2006). Property tailorability for advanced cvi silicon carbide composites for fusion. Fusion Eng. Des..

[50] Yamada R., Igawa N., Taguchi T., Jitsukawa S. (2002). Highly thermal conductive, sintered sic fiber-reinforced 3D-SiC/SiC composites: Experiments and finite-element analysis of the thermal diffusivity/conductivity. J. Nucl. Mater..

[51] Naslain R. (2004). Design, preparation and properties of non-oxide cmcs for application in engines and nuclear reactors: An overview. Compos. Sci. Technol..

[52] Jumel J., Lepoutre F., Enguehard F., Rochais D., Cataldi M. (2006). Microscopic thermal characterization of C/C composites. High Temperature Ceramic Matrix Composites.

[53] Zhang Q. (2009). Themophysical Properties and Microstructure Damage Characterization of C/SiC Composites. Ph.D. Thesis.

[54] Nie R., Jiao G., Wang B. (2009). Prediction on coefficient of thermal conductivity for 2D braided C/SiC composites. Acta Mater. Compos. Sin..

